# Dexmedetomidine as a useful adjunctive agent for preventing lung injury during emergence from anesthesia and tracheal extubation

**DOI:** 10.1186/s40981-025-00767-y

**Published:** 2025-02-01

**Authors:** Yasuhiro Watanabe

**Affiliations:** Department of Anesthesia, Japanese Red Cross Shizuoka Hospital, 8-2 Otemachi Aoi-Ku, Shizuoka, 420-0853 Japan

## To the editor:

I read a meaningful article addressing the effective use of a supraglottic airway during emergence from anesthesia in a patient with multiple giant bullae [1]. In thoracic surgery, there is no established method to minimize the risk of lung injury during emergence from anesthesia and tracheal extubation. By combining continuous infusion of dexmedetomidine and the method of tracheal extubation demonstrated by Arime et al. [1], I successfully managed a patient with a massive pulmonary air leak.

A 69-year-old man (height, 165 cm; weight, 63 kg; body mass index, 23.1 kg m^−2^) experienced a massive air leak on the second postoperative day after a right bullectomy and was scheduled for reoperation. The patient could not open his eyes due to systemic subcutaneous emphysema extending to the face and was emotionally disturbed and even depressed. Dexmedetomidine was used to prevent postoperative delirium and to facilitate a calm emergence from anesthesia.

An epidural catheter was inserted for postoperative analgesia, and general anesthesia was induced with fentanyl 100 μg, remifentanil 0.3 μg kg^−1^ min^−1^, and target-controlled infusion of propofol 2.5 μg mL^−1^. After neuromuscular blockade was achieved with rocuronium 60 mg, a 37-Fr left-sided double-lumen bronchial tube (Portex Blueline®, Smith Medical, Tokyo, Japan) was inserted. Continuous infusion of dexmedetomidine was subsequently started at 0.3 μg kg^−1^ h^−1^ without an initial loading dose to avoid adverse hemodynamic effects. General anesthesia was maintained with propofol 2.0 − 2.5 μg mL^−1^, remifentanil 0.1 − 0.15 μg kg^−1^ min^−1^, and epidural administration of levobupivacaine 20 mg by maintaining the bispectral index (BIS™, Medtronic, Minneapolis, MN, USA) at 40 − 60. A pneumostasis procedure was performed uneventfully using a polyglycolic acid mesh and fibrin glue. An i-gel™ (size 4) was inserted with a double-lumen tube in place; then, the double-lumen tube was removed under deep anesthesia. After confirming spontaneous breathing and response to verbal commands, the i-gel™ was removed without coughing or laryngospasm. Then, the dosage of dexmedetomidine was decreased to 0.25 μg kg^−1^ h^−1^ and was continued until the day after surgery. The operation time was 327 min and anesthesia lasted 439 min. The patient did not develop postoperative delirium and was appropriately sedated with a Richmond Agitation-Sedation Scale score between − 1 and 0. The postoperative course was uneventful, and the patient was discharged on the fifth postoperative day.

As in Arime et al.’s [1], our case was predisposed to serious lung injury during emergence from anesthesia and tracheal extubation. In a recent meta-analysis, dexmedetomidine was identified as an effective agent for preventing postoperative delirium in older patients [2]. Dexmedetomidine also possesses antidepressant properties [3], with confirmed efficacy in patients after cesarean section [4]. In the present case, perioperative infusion of dexmedetomidine was thought to have promoted uncomplicated tracheal extubation and contributed to the successful anesthetic management. Therefore, I recommend this approach for patients at an increased risk of lung injury during emergence from anesthesia and tracheal extubation.

## Data Availability

The data in this manuscript are available from the corresponding author upon reasonable request.
